# Medico-Chirurgical Transactions

**Published:** 1844-10

**Authors:** 


### Art. VIII.

*Medico-Chirurgical Transactions.* Vol. XXVI.—*London*, 1843. 8vo. Three Plates.

I. *Case of paralysis*, by John Webster, M.D. F.R.S. This is an interesting paper. The patient, a gentleman ætat. thirty-six, was for many months utterly unable to move, by voluntary effort, any muscle below the neck, excepting the diaphragm, while sensation remained perfect over the entire surface of the body, and the intellectual faculties and other senses continued unimpaired until death. The progress of the case was very slow, the disease advancing gradually to its climax; and the patient suffered much at times from convulsive spasms in the lower extremities. He had also occasional epileptic fits. The cuticular secretion was entirely suspended. The muscles of deglutition were unaffected throughout. On examination after death, no very remarkable appearances were detected in the cranium. The spinal theca, corresponding to the three or four lower cervical vertebræ, was much distended; and, on being cut into, the arachnoid cavity and sub-arachnoid tissue were found filled with lymph, which united the membranes to each other and to the cord, the adhesions being most firm on the anterior aspect. At this part the medulla was much diseased. Professor Todd, of King's College, examined this portion, and reports as follows: "I find great destruction (from softening) of the medullary substance of the posterior columns, especially that of the right side; the antero-lateral columns seem to have been also the seat of the softening process to a less degree, but I do not find that they have suffered any loss of substance. In examining the softened parts by the microscope, I detected very few of the proper nerve-tubes; and those which I did observe were much altered from their natural appearance; they had become opaque, and had assumed an indistinctly fibrous aspect. I was unable to find any trace of gray matter." The posterior roots of the nerves were unaffected. The preparation, it should be stated, had been preserved some time in spirits, before this examination was made by Dr. Todd.

It is needless to say how perplexing are morbid changes like these, when taken in connexion with the symptoms manifested during life. We remember a case that was brought under our notice some few years ago, the symptoms of which were precisely the converse of the above, viz. the persistence of voluntary motion, with the almost entire loss of sensation; and yet in this instance the morbid appearances were not very dissimilar. The gray matter was altogether destroyed, the interior of the spinal cord (in the lower cervical and upper dorsal regions,) being converted into a cream-like substance. Verily there are mysteries in these things which our philosophy has not yet unravelled.

II. *Case of bronchial calculus*, by Dr. S. C. G. Tice. The symptoms in this case did not lead to any suspicion of the true nature of the disease. They were pain in the right side, increased by pressure over the liver; dyspnea, which was latterly very urgent, amounting to a sense of suffocation upon lying down; the paroxysmal cough; great fetor of breath; deep-seated dysphagia; and ultimately laryngitis. Death occurred suddenly, after an illness of six weeks. The physical signs of disease in the chest were entirely absent. On examination, there was found an abscess, of the size of a pullet's egg, in a mass of enlarged bronchial glands, at the bifurcation of the trachea. It opened into both bronchi and the œsophagus; and contained a quantity of calcareous matter, some of which was very hard, and some of a soft consistence. A hard triangular portion was firmly wedged in the aperture communicating with the right bronchus. It was found to consist of phosphate of lime.

III. *On congestive pneumonia, consequent upon surgical operations, diseases, and injuries*, by Mr. J. E. Erichsen. The form of disease noticed in this communication deserves especial attention, not less from its frequency, than from its very insidious character. Out of sixty-two cases of death after surgical operations and injuries (exclusive of burns), taken indiscriminately from the records of University College Hospital, in no less than twenty-eight there were evident signs of pneumonia, as shown either by the diseased condition being confined to one lung, by its having advanced to solidification, or by the coexistence of marks of inflammatory action in the pleuræ or bronchial mucous membrane. In eleven the lungs were in a doubtful condition, presenting the characters which are common to the first stage of pneumonia and to passive congestion, without there being any collateral circumstances by which the diagnosis could be more clearly established. In nine the lungs were found more or less diseased, but not inflamed or congested; and in fourteen only were they quite healthy. The appearances observed in the affected organs were precisely those which distinguish what is generally denominated *typhoid pneumonia;* and it is a remarkable fact with regard to the location of the disease, that in twenty-two of the cases it existed in both lungs together, though not to the same degree—the right and left lungs being each the sole seat of disease in only three cases. Our readers will at once perceive how very different is this state of things from that which prevails in the more common form.

IV. *Researches into the connexion existing between an unnatural degree of compression of the blood contained in the renal vessels, and the presence of certain abnormal matters in the urine*, by Mr. G. Robinson. This is an account of two series of experiments performed upon the kidneys of rabbits; from the first of which it appears that, when the return of blood is either entirely or partially prevented by ligature of the renal vein, the urine, examined at various periods, from three minutes and half to two days and half, will be found to contain albumen, fibrin, or blood, in variable quantities. In the second series similar results were produced by removing one kidney and tying the abdominal aorta below the point where the renal arteries are given off. We very much doubt the value of these vivisections in determining the point for which they were undertaken, viz. the ascertaining "of the precise cause of the appearance of albumen in the kidney," and, we believe, the rationale of inflammation; for it is quite clear that, excepting in very extraordinary cases, such an amount of *sudden* compression as was produced in them (and which certainly appears to be an essential condition) cannot take place in the human body. And although, in the instances before us, the results were evidently effected by merely mechanical means, is this any proof that in the slower or more complicated actions of inflammation or other disease, we have to look for no further cause?

V. *An account of an unusually large biliary calculus voided from the rectum*, by J. A. Wilson, M.D. The calculus in this case was as large as a full-sized walnut. On its passage to the bowels it induced jaundice, but occasioned *no pain.* The centre consisted of nearly pure cholesterine, and the external part of inspissated and altered bile, with a little cholesterine or fatty matter.

VI. *On fatty degeneration of the arteries*, by G. Gulliver, Esq. F.R S. From the researches of this gentleman it appears, 1, that the white or buff-coloured opaque spots of the inner membrane of the arteries are of a fatty nature; 2, the soft matter, which has been generally called atheroma, and which often collects between the inner and middle coats, is also fatty; 3, the fatty matter is frequently found in the substance of both these coats; 4, a fatty degeneration of the tunics of the arteries is generally connected with that state of them which is the most frequent cause of aneurism, as well as of their obstruction, occlusion, or wasting in aged people; 5, the matter usually contains cholesterine and oleine, and often some margarine; 6, the tunics of ossified arteries, as well as the bony plates, are often pervaded by the fatty substances just mentioned. In an appended note some valuable observations are made upon fatty degeneration in other organs, as the testis, the kidneys, the lungs, &c. which—as indeed the whole paper—deserve a most attentive perusal, but can be only alluded to in this place.

VII. *Remarks on the calculi in St. George's Hospital*, by Dr. H. Bence Jones. This paper is already so condensed that we are unable to compress it into smaller compass.

VIII. *Case of ulceration of the internal jugular vein, communicating with an abscess*, by Mr. W. Bloxam. The abscess resulted from suppuration of a gland under the angle of the jaw, as a sequela of scarlatina. It opened spontaneously, and the bleeding commenced five days afterwards. The ulcer was on the inner side of the vein, of an oblong shape, and about five lines in its long axis.

IX. *Account of an hysterical affection of the vocal apparatus, with cases*, by Mr. Oscar Clayton. The cases occurred, at three separate periods, in a charitable institution for the maintenance and education of female children from the age of nine to fourteen. In the first attack there were well-marked pyrexial symptoms at the commencement, but in the subsequent ones the hysterical and imitative character developed itself at once. The sounds produced by the different patients were exceedingly various, and very loud, beginning in all with a constant hacking cough. No means of treatment was of any avail, until the children were completely separated from each other.

X. *Case of erectile tumour in the popliteal space*, by Mr. Liston. This is interesting, from its rarity in such a situation. It was removed with perfect success. Appended to this paper is a note of an anomalous growth which the author removed some years ago from the sterno-mastoid muscle.

XI. *Two cases of osteo-sarcoma of the thigh-bone, requiring amputation of the limb in both instances*, by Mr. Frogley.

XII. *Remarks on cancrum oris*, by Dr. H. Hunt. The object of this communication is to point out the efficacy of the chlorate of potash in arresting the progress of this frightful disease, or even effecting a cure, if it has not advanced too far. The quantity of the salt given varies from twenty to sixty grains, according to the age of the child, in divided doses, in twenty-four hours, dissolved in water. The beneficial effect is often observed on the following day, almost always on the second: the disagreeable fetor soon lessens, the sores put on a healthy reparative action, the dribbling of saliva diminishes; if there is mere ulceration it very speedily heals; if there is an eschar, it soon separates; and the sore granulates kindly. Aperients are frequently requisite at the same time.

XIII. *Case of ulceration of the pulmonary artery into an abscess of the lungs*, by W. Crowfoot, Esq. The patient was a medical man, affected with tubercular disease of the left lung. The *active* hemorrhage, which destroyed him, commenced about a month before death. On examination after death, the upper part of the lung was found entirely occupied by a large cavity, containing about half a pound of grumous and coagulated blood. The pulmonary artery was so large as at first to be taken for the aorta, for it had a complete curvature to the right, as high up and upon a line with the clavicle of the left side. It opened into the abscess by a funnel-shaped orifice, about two inches from its bifurcation.

XIV. *Cases of strangulated hernia, reduced "en masse,"* by Mr. Luke. The accident which forms the subject of this communication is one little noticed by British writers on surgery; but, when it does occur, is of such grave importance as well to merit the most serious attention. It consists in the reduction of the hernial tumour through the aperture of the abdominal parietes, together with its investing sac, so that the contents still remain strangulated, and as imperatively demand liberation as if the tumour was unremoved from its original situation. It is generally caused by the employment of too much force in the use of the taxis. In the instances, however, which occurred in Mr. Luke's practice, this had not been the case, no violent efforts having, apparently, been had recourse to. We have not space to enter upon any examination of these cases, but must confine ourselves to a brief sketch of the method of proceeding which should be adopted when the occurrence of such a serious accident is suspected. Of course, before determining upon any decided step, the most rigid manual examination must invariably be instituted ; and while conducting this the surgeon will bear in mind the usual condition of parts when a hernia has been regularly reduced. He will remember that the presence of the sac, even without any contents, causes an abnormal fulness, obscures the spermatic cord, and renders the edges of the abdominal aperture somewhat indistinct. If, therefore, upon examination, he finds the contrary of all this—no fulness of parts, a well-defined cord, and a large aperture free and unobstructed, and with unobscured margins, his suspicions will be greatly strengthened. In some cases there may, in addition, be circumscribed pain in the situation of the returned herniæ ; and it is possible that a tumour may be indistinctly felt deep in the abdomen. But whether this be so or not, it is right, if the former circumstances exist, to endeavour to accomplish re-protrusion, by causing the patient to be placed in the erect posture, and then to cough forcibly, strain, and make exertion. If these means are successful—and they may be when the hernia is either in or very near the internal aperture—the ulterior course to be produced is at once self-evident. If they fail, Mr. Luke advises an exploratory operation to be performed ; and the following is the one he recommends: A free opening should be made through the integuments, so as to expose the tendon of the external oblique, the external abdominal ring, and the cord ; the condition of these parts being carefully noted, either as confirming or setting aside the former opinions. The next step is to lay open the inguinal canal; the state of the cord will then be ascertained, and the surgeon should also seek for the condensed cellular capsule which usually invests the hernial sac, and remains in its place even when the sac is returned. If no tumour is now discovered, the finger should be carried through the internal ring, enlarging it if necessary, and passed from side to side. If a hernial tumour be present it will be at once recognized and found lying externally to the general peritoneal membrane, although within the parietes, and presenting a rounded surface and tense feel. Should the tumour not be present, the circumstance may be ascertained by observing the smooth surface of the peritoneum, and the continued adhesions which it maintains with the parietes immediately surrounding the ring. When a tumour is discovered, it should be brought into the canal by incising the ring to the requisite extent, and should then be opened, and the neck of the sac freely divided. Great care should also be taken that the contents of the sac be perfectly liberated, and, for this purpose, the finger should be passed through the neck after they are reduced.

XV. *Observations on the medicinal properties of the cannabis sativa of India*, by J. Clendinning, M.D. The author gives a very favorable account of this drug. Its exhibition was followed, with remarkably few exceptions, "by manifest effects as a soporific or hypnotic in conciliating sleep, as an anodyne in lulling irritation, as an antispasmodic in checking cough and cramp, and as a nervine stimulant in removing languor and anxiety." These effects were observed in both acute and chronic affections, and in patients of all ages and both sexes. It had also the advantage of not producing the injurious effects of opium. We. think the remedy well deserving further trial.

XVI. *On the sugar of diabetic blood*, by Dr. H. Bence Jones. This is an account of the application of H. Trommer's new method of distinguishing between cane and grape sugar, to the detection of sugar in diabetic blood. The test consists in the perfect solution of the precipitate which first forms when caustic potash is added in excess to a solution of sulphate of copper and grape sugar, and the after-formation of a peculiar coloured precipitate by heating the mixture. In performing this experiment with serum, it is essential that the albumen be entirely removed by evaporation to perfect dryness; the residue must then be reduced to a very fine powder, and treated with water for a considerable time: then filtered fluid will sometimes, but not always, give the desired results.

XVII. *Observations on encysted hydrocele*, by R. Liston, Esq. The object of this paper is to show that some of these collections of fluid are more intimately connected with the testicle or its seminiferous tubes than has been generally supposed. He was first led to form this opinion from finding the fluid drawn from a sac of this nature "quite full of spermatozoa," and containing also some of the primitive cells in which the spermatozoa are developed, and a certain number of mucous globules. If further observation should confirm these views, the difficulty of effecting a cure will be at once explained.

XVIII. *Account of an epidemic at Teheran*, by Dr. C. W. Bell. In this very singular epidemic, which occurred in January and February, 1842, the patients were affected, often suddenly, with numbness of one side of the body, spasms, convulsions, and insensibility; these were accompanied with extreme excitement of the circulation, violent headach, and frequently pain in the region of the heart. The disease was evidently periodical. Venesection and all lowering remedies were found to be injurious; but a cure was generally effected by the use of assafœtida, in the form of enema, and full doses of carbonate of iron by the mouth. Quinine did not prove trustworthy.

XIX. *On the nature of the ossification of encysted tumours*, by J. Dalrymple, Esq. On examining, by the microscope, a small tumour which had been removed from the tarsal cartilage, he found that its concentric layers were entirely composed of epithelium scales closely agglutinated together; but instead of the usual transparent thin lamina with its central nucleus, they were thickened and hard, and contained granular earthy molecules, which could be removed by immersion in weak muriatic acid. No amorphous earthy deposit existed around or among the scales. The central nuclei were clear and large.

XX. *On the anatomical characters of some adventitious structures*, by Dr. Hodgkin. The design of this very valuable communication is to demonstrate the following points: That the type of the whole family of cancerous diseases is that of compound serous cysts; that microscopic examination does not furnish perfectly conclusive tests of any particular form of adventitious structure to which a specimen may belong, but that it demonstrates the application of the nucleated-cell theory, whilst it is fatal to that of cancerous matter being formed in the blood, and eliminated at the spots at which the tumours became manifest; that to have a complete view of the mode of production of these structures, we must combine the cell-theory of Schwann and Müller, the coagulation-principle which the author had previously suggested, and the process of organization investigated by Kiernan, and that none of these phenomena, taken singly, is an adequate test of malignancy; that chemical analysis affords an imperfect and inadequate criterion; that in removing a tumour of this class, it is extremely important to leave behind none of those minute cysts which often form granules in the surrounding cellular membrane, though it may appear perfectly healthy in other respects; and, lastly, that the infiltrated form of these diseases occurs in the neighbourhood of the purely adventitious growth, when these structures have been the seat of inflammation, and that the chances of success from operation are consequently infinitely diminished when such inflammation has taken place. The presence of the peculiar matter of the disease in the interior of vessels appears to be one of the modes in which infiltration, the result of inflammation, exhibits itself, and is therefore not a valid argument in favour of the preexistence of such matter in the circulating blood. We feel that it is quite unnecessary to say one word regarding the importance of these subjects, and shall simply but earnestly counsel our readers to give themselves the pleasure and profit of a careful perusal of the original, which would be only marred and mutilated by any attempt at condensation.

XXI. *Case of a foreign body in the right bronchus*, by Sir B. C. Brodie. The details of this case, in which the celebrated Mr. Brunel was the sufferer, are sufficiently familiar; we shall merely mention that the foreign body, a half sovereign, was successfully removed (after an opening had been made in the trachea, and the forceps employed without avail), by placing the patient on an apparatus by which his body was inverted, and striking the back over the bronchus with the hand; two or three efforts to cough dislodged the coin and it fell into the mouth. The same plan had been tried before the tracheal opening was made, but it produced such distressing feelings of suffocation that the attempt could not be persisted in.

XXII. *On the pathology of the ear*, by Mr. J. Toynbee. This is the sequel of a paper which we formerly noticed with praise, (Br. and For. Med. Rev., vol. XIV, p. 62,) and we are well pleased to find the author still persisting in a course which must eventually lead to valuable results. He has now performed 120 dissections, and the more extended experience thus acquired fully bears out the opinions formerly stated as to the great frequency of disease of the tympanic cavity. The present communication is occupied with the subject of inflammation of the mucous lining of the tympanum, in which he recognizes three stages. In the *first* stage, the membrane retains its natural delicacy of structure, but the blood-vessels are considerably enlarged and contorted, and blood is effused into its substance, or more frequently at its attached surface. The *second* stage is characterized by a variety of pathological phenomena, of which the principal are: 1. A considerable thickening of the membrane, which is often pulpy and flocculent. 2. Concretions of various kinds on the surface of the thickened membrane. 3. The membranous bands of which notice was formerly taken. In the *third* stage the membrane is ulcerated, the membrana tympani destroyed, and the tensor tympani muscle atrophied. The ossicula are diseased and discharged from the ear, and not unfrequently the walls of the tympanum become affected and the brain involved. Now out of the whole number of dissections, the first stage of inflammation existed in 20, the second in 65, the third in 6, and the organ was found healthy in 29 only.

XXIII. *On the effects of rickets on the growth of the skull*, by Alex. Shaw, Esq. An interesting paper. It is well known that in patients affected with this disease, the body generally retains the characteristics of childhood, the upper half bearing an undue proportion in the lower. Mr. Shaw has shown that the same obtains with the cranium as compared with the face, in the natural skull, the ratio of the former to the latter being as 6 : 1, while in the rickety it is as $7\frac{1}{13}$ : 1. He explains this upon the principle, that as years advance the growth of the face proceeds with far greater rapidity than that of the cranium, and consequently, when from disease the progress of development is arrested, that part will most suffer which in ordinary cases would have gained the most decided increase. In confirmation of this, he has observed in individuals whose general growth is above that of the standard size, the dimensions of the skull show a precisely opposite arrangement, the cranium bearing to the face the proportion of only 5 : 1. The diminished or enlarged size of the face appears to depend upon the greater or less development of the frontal and maxillary sinuses; and it is a remarkable fact connected with this, that the diameters of the orbits do not vary, being the same in the normal, the rickety, and the gigantic skull. In reference to the maxillary bones, he has also been led to the opinion that the part which suffers most in rickets, is that portion which is appropriated to the permanent molares.

XXIV. Mr. Lloyd has narrated three cases in which spermatozoa was found in the fluid of *ordinary hydrocele*. Is there no fallacy in these cases?

XXV. *Statistics of Bethlem Hospital, with remarks on insanity*, by Dr. Webster. The following table is very interesting in many respects, exhibiting as it does the number of patients who were admitted, discharged cured, or died, during five periods of twenty years each, ending the 31st December respectively:

| In 20 years ending | Number admitted. | Number cured. | | | Number died. | | |
|---|---|---|---|---|---|---|---|
| 1762 | 3286 | 1069 | or $32\frac{1}{4}$ | per cent. | 714 | or $21\frac{2}{3}$ | per cent. |
| 1782 | 3945 | 1366 | $34\frac{1}{2}$ | ,, | 560 | $13\frac{1}{5}$ | ,, |
| 1802 | 3906 | 1379 | $35\frac{1}{3}$ | ,, | 203 | $5\frac{1}{5}$ | ,, |
| 1822 | 2149 | 892 | $41\frac{1}{2}$ | ,, | 111 | $5\frac{1}{6}$ | ,, |
| 1842 | 4404 | 2269 | $51\frac{1}{2}$ | ,, | 224 | $5\frac{1}{14}$ | ,, |
| Totals . . | 17,690 | 6975 | $39\frac{1}{3}$ | ,, | 1812 | $10\frac{1}{4}$ | ,, |

We would especially direct attention to the very gratifying diminution of mortality and increased number of cures during the later periods, in which milder and more humane methods of treatment have been in operation. Suicides have likewise become more rare; from January 1750 to January 1770 they occurred in the proportion of 1 in about 202; during the last twenty years the ratio has been 1 in 925. During the twenty years ending January 1770, 1 patient out of every 66 made his escape; during the last twenty years the proportion has been 1 in 292, though restraint is now the exception instead of the rule.

There have been 72 dissections made since the 1st January, 1837, of patients who died in the hospital. In 59 of these there was infiltration of the pia mater; in 59, turgidity of the blood-vessels of the brain and membranes. In 41, dropsy of the ventricles. In 27, water at the base of the brain. In 19, bloody points on the cut surfaces of the medullary substance. In 16, thickening and opacity of the arachnoid. In 14, the colour of the medullary or cortical substance was brown, pink, gray, violet, ochre, or white. In 13, there was effusion of blood in the brain. There were also various other lesions in some patients, as effusion of pus, altered consistence, dryness of the membranes, &c. In 55 of the 72 cases there was also disease in the thoracic organs.

The volume is altogether one of unusual interest.

